# Excising the neovagina due to introital atresia and closed neovaginal loop after sigmoid vaginoplasty: A case report

**DOI:** 10.1097/MD.0000000000024972

**Published:** 2021-03-05

**Authors:** Rui Wang, Qi Su, Zhaopeng Yan

**Affiliations:** a Department of Critical Care Medicine; b Department of General Surgery, Shengjing Hospital of China Medical University, China.

**Keywords:** case report, closed neovaginal loop, introital atresia, sigmoid vaginoplasty, vaginal agenesis

## Abstract

**Introduction:**

Vaginal agenesis is a congenital disorder, which can be managed by nonsurgical dilation or surgical reconstruction of the vagina. The sigmoid vaginoplasty procedure is a popular approach, which pulls down part of the sigmoid colon to form a neovagina. One complication of this procedure is introital stenosis.

**Patient concerns:**

A 55-year-old woman presented to the outpatient general surgery department with severe, persistent abdominal pain. The patient was diagnosed with congenital absence of uterus and vagina, and a sigmoid vaginoplasty was performed 34 years ago.

**Diagnosis:**

A pelvic MRI and an abdominal enhanced CT scan were performed, finding that the uterus was absent, and the os of the vagina was closed, forming a closed loop full of fluid. Introital atresia and closed loop of neovaginal colon conduit were diagnosed.

**Interventions:**

Based on our conclusions and the patient's consent we surgically removed the neovagina.

**Outcomes:**

After surgery, the abdominal pain was relieved, and the patient reported full recovery during a 6-month follow-up appointment.

**Conclusion:**

Introital stenosis is one of the long-term complications of sigmoid vaginoplasty procedure. Introital stenosis, leading to introital atresia, is rare but may occur. Surgical removal of neovagina can relieve the pain in patients who do not have the demand of sexual intercourse.

## 1 Introduction

Vaginal agenesis is a congenital disorder. The sigmoid vaginoplasty procedure is one of the most popular approaches for vaginal agenesis patients. The long-term complications of sigmoid vaginoplasty procedure typically include introital stenosis, malignant degeneration, inflammatory bowel disease, and fistulae.^[1]^ Here, we submit a previously unreported complication of sigmoid vaginoplasty. We report a case of a 55-year-old female patient who presented with severe chronic lower abdominal pain caused by introital atresia and a closed neovaginal loop. Surgical removal of the neovagina relieved the pain.

## 2 Case presentation

A 55-year-old woman presented to the outpatient general surgery department with severe, persistent abdominal pain, which had worsened over the past 6 months and could not be managed with over-the-counter pain medication. In reviewing the patient's medical history, we found that the patient was diagnosed with congenital absence of uterus and vagina, and a sigmoid vaginoplasty was performed 34 years ago. After the procedure, the patient was sexually inactive and did not complete a dilation protocol. About 5 years ago, the patient suffered acute lower abdominal pain and high fever. In the process of admission to the hospital, the patient felt weak and fell to the ground, landing heavily on her abdomen. The patient reported a tearing pain in the lower abdomen. Afterwards, massive purulent fluid was discharged from the vagina and the severe abdominal pain and fever were relieved in the next few hours. The patient did not receive further evaluation or treatment and left the hospital. About half a year ago, the patient felt the abdominal pain again and come to us seeking for medical assistance.

**Figure 1:**
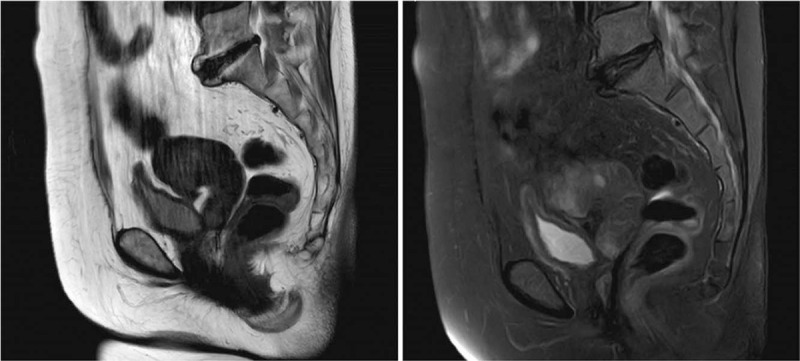
A pelvic MRI showing absence of the uterus, and closure of the os of the vagina, forming a closed loop full of fluid.

A pelvic MRI and an abdominal enhanced CT scan were performed, finding that the uterus was absent, and the os of the vagina was closed, forming a closed loop full of fluid (Fig. 1). We concluded that the pain might be caused by the closed loop of the vagina. As seen in introital atresia, the mucus secreted by the neovagina (which was originally part of the sigmoid colon) filled the cavity over time and created increasing amounts of pressure and tension, leading to abdominal pain. Based on our conclusions and the patient's consent we surgically removed the neovagina. After surgery, the abdominal pain was relieved, and the patient reported full recovery during a 6-month follow-up appointment.

## 3 Discussion

Vaginal agenesis is a congenital disorder defined as the congenital absence of the vagina with variable uterine development. It is also called Mayer–Rokitansky–Küster–Hauser (MRKH) syndrome, which is caused by agenesis or hypoplasia of the Müllerian duct system. The etiology of MRKH is unknown. The prevalence for vaginal agenesis is 1 in 5000.^[1]^ In MRKH patients, the karyotype analysis shows a normal female karyotype pattern and the ovaries in normal condition, indicating normal female secondary characteristics. However, vaginal agenesis is usually accompanied by cervical and uterine agenesis. The absence of a uterus can lead to amenorrhea, which is the most common symptom of this disorder.^[2]^

The most common nonsurgical treatment for vaginal agenesis is the use of vaginal dilators. The self-dilation treatment is recommended by the American College of Obstetricians and Gynecologists.^[3]^ Nonsurgical vaginal elongation by dilation is advised as the first-line approach, as most compliant patients have achieved anatomic and functional success.^[4]^ If this is not a viable option, surgery is recommended. Sigmoid vaginoplasty is one surgical option, while other choices include the McIndoe procedure, Williams vaginoplasty, the Davydov procedure, and a modified Vecchietti procedure. The sigmoid vaginoplasty procedure involves removing part of the sigmoid colon with blood supply. An end-to-end anastomosis is then performed to recreate a patent colonic tract. The excised colon with blood supply is then fixed to the vaginal pit incision to form a neovagina.

Thirty four years ago, sigmoid vaginoplasty was selected and performed on this patient. When the patient presented to us, no medical records were found. However, the abdominal CT scan and pelvic MRI confirmed the medical history of sigmoid vaginoplasty and the diagnosis of introital atresia.

The long-term complications of sigmoid vaginoplasty procedure include introital stenosis, mucosal trauma, Diversion colitis, inflammatory bowel disease, fistulas, and adenocarcinoma of the segmental colon.^[5]^ Introital stenosis, leading to introital atresia, is rare but may occur.^[5,6]^ As the patient had not had sexual intercourse since the procedure 34 years ago, the introital atresia was not discovered. Due to introital atresia, the neovagina formed a closed loop, allowing for mucus secreted by the segmental colon to build up. As the pressure in the loop increases, pain occurs. The pain was relieved when the neovagina was removed.

This situation is unusual, and in most cases of lower abdominal pain, other causes should be considered. Simply removing the neovagina may not always be the correct action. Due to the reported medical history from 5 years ago that similar abdominal pain was relieved by an accidental fall and the subsequent nonsurgical neovaginal drainage, we correctly concluded that the abdominal pain could be treated by removing the neovagina. This situation is relatively rare, since cases of vaginal agenesis are uncommon.

## Acknowledgments

We would like to thank Editage (www.editage.com) for English language editing.

## Author contributions

**Conceptualization:** Zhaopeng Yan.

**Funding acquisition:** Rui Wang.

**Investigation:** Qi Su.

**Supervision:** Zhaopeng Yan.

**Writing – original draft:** Rui Wang.

**Writing – review & editing:** Qi Su.
